# Fluoride-modified implant surfaces improves osseointegration in the tibias of rats with induced diabetes

**DOI:** 10.1590/0103-6440202305439

**Published:** 2023-12-22

**Authors:** Guilherme José Pimentel Lopes de Oliveira, Lucas Amaral Fontanari, João Antônio Chaves de Souza, Rubens Spin-Neto, Carlos Nelson Elias, Elcio Marcantonio, Silvana Regina Perez Orrico

**Affiliations:** 1 Department of Periodontology, UNESP - Univ. Estadual Paulista, Araraquara Dental School, Araraquara, São Paulo, Brazil.; 2 Department of Odontology, UFU - Univ. Federal de Uberlândia, Uberlândia, Brazil.; 3 Department of Odontology, UFG - Univ. Federal de Goiás, Goiânia, Brazil.; 4 Department of Dentistry - Oral Radiology, Aarhus University, Aarhus, Denmark.; 5 Biomaterials Laboratory, Instituto Militar de Engenharia, Rio de Janeiro, Rio de Janeiro, Brazil.; 6 Advanced Research Center in Medicine , Union of the Colleges of the Great Lakes (UNILAGO), São José do Rio Preto, Brazil.

**Keywords:** Diabetes Mellitus, implant surfaces, osseointegration

## Abstract

This study evaluated the influence of a fluoride-modified titanium surface on osseointegration in rats with induced diabetes. One hundred and eighty rats were randomly allocated into 3 groups with 60 animals each: Control group (C): Animals without diabetes; Diabetes Group (D): Animals with uncontrolled induced diabetes; Controlled Diabetes Group (CD): Animals with diabetes induced controlled by the insulin administration. Diabetes was induced by streptozotocin injection. Each animal received 2 implants in the proximal tibial metaphysis, one with the machined surface (M) and the other one with a fluoride-modified titanium surface (F), after 4 weeks of induction of diabetes. The animals were submitted to euthanasia 2, 4, and 6 weeks after the implant placement (n = 20 animals/group). The osseointegration was evaluated by the implant removal torque test and the histometric analysis of the non-decalcified histological sections: 1) Contact bone/implant (%BIC); 2) Bone tissue area between implant threads (%BBT). Implants with F surface showed a higher removal torque than implants with surface M in all groups. There was no difference in %BIC between the groups regardless of the surface used. The F surface showed a tendency to present higher %BBT values for the 3 evaluation periods in the D group. The fluoride-modified implant surface has no impact on the %BIC and %BBT. However, the fluoride-modified implant surface increases the locking of the implants with the bone. The hyperglycemia was associated with lower removal torque values despite the surfaces of the implant used.

## Resumo

Este estudo avaliou a influência de uma superfície de titânio modificada com flúor na osseointegração em ratos com diabetes induzida. Cento e oitenta ratos foram distribuídos aleatoriamente em 3 grupos com 60 animais cada: Grupo controle (C): Animais sem diabetes; Grupo Diabetes (D): Animais com diabetes induzida descompensada; Grupo Diabetes Controlado (CD): Animais com diabetes induzido controlado pela administração de insulina. O diabetes foi induzido por injeção de estreptozotocina. Cada animal recebeu 2 implantes na metáfise proximal da tíbia, um com superfície usinada (M) e outro com superfície de titânio modificado com flúor (F), após 4 semanas de indução do diabetes. Os animais foram submetidos à eutanásia 2, 4 e 6 semanas após a colocação do implante (n = 20 animais/grupo). A osseointegração foi avaliada pelo teste de torque de remoção do implante e pela análise histométrica dos cortes histológicos não descalcificados: 1) Contato osso-implante (%BIC); 2) Área de tecido ósseo entre as roscas do implante (%BBT). Os implantes com superfície F apresentaram maior torque de remoção do que os implantes com superfície M em todos os grupos. Não houve diferença no %BIC entre os grupos independente da superfície utilizada. A superfície F mostrou tendência a apresentar maiores valores de %BBT para os 3 períodos de avaliação no grupo D. As superfícies de implantes modificadas com flúor não influenciaram nos dados de %BIC e %BBT. Entretanto, essas superfícies aumentaram o travamento dos implantes no tecido ósseo. A hiperglicemia foi associada a menores torques de remoção dos implantes independentemente do tipo de superfície de implante utilizada.

## Introduction

The use of dental implants has been increasingly common for fully and partially edentulous patient rehabilitation with high survival rates and long-term success ^1^,^2^). However, it has been mentioned that some host conditions can influence the occurrence of early and late failures on the osseointegration ^3^, thus placing these patients at risk of not obtaining a good clinical outcome of the oral rehabilitations supported by implants.

Among these conditions, Diabetes mellitus deserves to be highlighted since it is a disease of high prevalence and has been considered a major risk factor for osseointegration failure ^4^,^5^. Regardless of the type of diabetes that can affect patients, this disease is characterized by peaks of hyperglycemia that are related to the formation of advanced glycation end products (AGEs), microvascularization problems, and impaired the collagen maturation that can induce systemic complications, including on bone tissue turnover ^5^. Diabetic patients are at greater risk of tooth loss due to caries and periodontal disease, which would put them under a greater chance of being rehabilitated with dental implants ^6^,^7^; however, it has been also reported that these patients present lower success and survival rates of the implants ^8^.

As a way to improve predictability and accelerate the process of osseointegration, modifications on the dental implant surface have been proposed at the level of macrostructure and microstructure design ^9^,^10^. The modifications of microstructure are related to the acceleration of obtaining the secondary stability due to the stimulus in the process of osseointegration ^9^. In general, the implant's surface modifications are related to the chemical composition or surface roughness that can increase the adhesion of undifferentiated mesenchymal cells and blood elements that will induce the formation of bone in an early phase of osseointegration ^11^. 

Among the techniques used to modify the implant surfaces, the fluoride-modified surfaces is a promising technique since this modification has been related to the enhance of the nanoroughness ^12^, stimulating osteoblast proliferation and differentiation ^12^,^13^, enhancing the bone formation surrounding the implants at early healing stages^14^,^15^, up-regulated the expression of important bone formation markers as the collagen type 1, osteocalcin, osteoprotegerin and alkaline phosphatase ^13^-^15^, and reduce the effect of the *Porphyromonas gingivalis* on the adhesion of the osteoblast on the implant surfaces ^16^. Clinical studies have shown that the use of fluoride-modified surface implants presents high predictability in implant-supported rehabilitation therapy ^18^,^19^. In a prospective clinical study where 125 implants were installed in fully edentulous mandibles in 25 patients, Collaert et al. ^(^ ^17^ demonstrated that fluoride-modified surface implants had survival rates of 100% with a mean bone loss of 0.1 mm after two years of follow-up. Mertens and Steveling ^18^ evaluated prospectively 43 implants with fluoride-modified surfaces that were placed in 15 patients after 5 years of follow-up. It was shown that these implants presented a survival rate of 97% and a mean peri-implant bone loss of 0.1 mm.

Therefore, it is plausible to hypothesize that implants with fluoride-modified surfaces would be useful in cases in which bone formation and remodeling are impaired, as seen in patients with poorly controlled diabetes. Then, this study aimed to evaluate the effect of the fluoride-modified implant surfaces on osseointegration in tibias of rats with induced diabetes, testing the hypothesis that the beneficial effect of these surfaces would trespass the challenges of an impaired bone formation and remodeling that occurs in hyperglycemic conditions.

## Material and methods

### Ethical Considerations

A total of 180 male Wistar rats with a mean body weight of 200g at the beginning of the study were used. The animals were kept in polypropylene boxes (n = 4 animals per box at maximum), in an environment with temperature, humidity, and controlled light, with access to rat chow and water *ad libitum* at the vivarium of the Araraquara School of Dentistry - UNESP. All procedures to which the animals were submitted were previously approved by the Ethics Committee on the Use of Animals of our institution (Proc. CEUA No. 10/2011), in compliance with the applicable ethical guidelines and regulations of the International Guiding Principles for Biomedical Research Involving Animals. All recommended Animal Research Reporting In Vivo Experiments guidelines were followed in this study.

### Groups and study design

**Figure 1:**
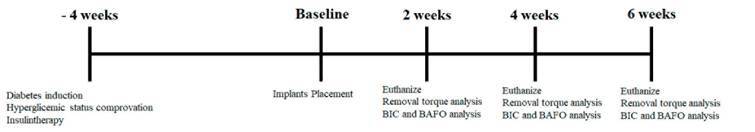
Flowchart of the experiment.

The animals were randomly allocated in three groups of 60 animals each according to the systemic condition of the animals: Control group (C): Animals without diabetes; Diabetes Group (D): Animals with uncontrolled induced diabetes; Controlled Diabetes Group (CD): Animals with induced diabetes treated with insulin. Four weeks after the induction of diabetes, all the animals were submitted to the random installation of one type of implant with different surfaces in the proximal tibial metaphysis of both tibias: Machined (M) and fluoride-treated surface (F). The animals were followed for 3 experimental periods of 2, 4, and 6 weeks after implant placement (20 animals per group/period). Ten of the animals of each group were used to perform the biomechanical analysis while the other 10 animals were used to perform the histometric analysis (Figure 1). The histometric analysis of the bone-implant contact (%BIC) was considered the primary variable of this study. The sample size calculation was based on a previous pre-clinical study that evaluated the osseointegration of different implant surfaces in hyperglycemic rats ^(^ ^19^. The minimum difference in the averages of the %BIC considered statistically significant between the groups was 18.1 % with the mean of the standard deviation of 12.2 %. Establishing the type I error of 0.05 and the beta power of 0.80 the minimum sample size of the groups must be 10.

### Induction of diabetes mellitus and glycemic control

After a 16-hour fasting period but with water *ad libitum*, 50 mg of streptozotocin (STZ) per kg of body weight dissolved in citrate buffer pH 4.5 was administered intraperitoneally in animals of the D and CD groups. Feeding was restored to animals 1 hour after drug administration. The animals in Group C received the same treatment protocol, but only with saline instead of streptozotocin solution.

After 24 hours of the streptozotocin injections, the hyperglycemia was proved by the analysis of the glycemic level. The blood collection for glycemic level determination was performed by cutting approximately 2 mm from the distal end of the tail of each animal, which remained in a heated box for 2 minutes for vasodilation. The blood samples were collected in Eppendorf tubes containing liquemine (Roche, São Paulo, Brazil). After the blood centrifugation (2500 rpm for 10 minutes) to obtain the plasma, the glycemic test was carried out by the enzymatic glucose-oxidase method (Colorimetric Glucose Kit PAP Liquiform - Labtest Diagnostica SA, Lagoa Santa, Brazil) at the laboratory of clinical Biochemistry of the Faculty of Pharmaceutical Sciences at Araraquara - UNESP. The glycemic level was evaluated weekly throughout the experiment. As inclusion criteria, the rats should present glycemic levels above 300 mg/dl after 24 h of diabetes induction.

Regarding the glycemic control in the CD group, these animals were treated twice daily (8:00 and 18:00 hours) with 2.5 U to 3 U of NPH Insulin (Humulin NPH U-100 - Lilly) shortly after the confirmation of the hyperglycemic state, which continued throughout the experimental period. The insulin was administered subcutaneously in 0.3 ml. The animals in Groups C and D received daily injections of 0.9% sodium chloride with the same volume received by the CD group. The time interval and route of administration were also equal to those of the insulin applied in the DC Group, throughout the experimental period. To guarantee the glycemic condition in each group, animals with glycemic levels lower than 180 mg/dl were kept in the CD group, while glycemic levels higher than 300 mg/dl were used to keep the animals in the D group.

### Surgical Procedure

The animals were anesthetized with a combination of Ketamine (0.08mL / 100g-Cetamin®-Syntec do Brasil Ltda, Santana do Parnaíba, Brazil) and Xylazine Hydrochloride (0.04mL / 100g-Xilazin® Syntec do Brasil Ltda, Santana do Parnaíba, Brazil), intramuscularly. Then the trichotomy of the internal region of the hind paws just below the knee was performed and the antisepsis with 70% iodinated alcohol solution was made. A 3-cm incision was made with a number 15 scalpel blade, and then the soft tissue was detached to expose the bone tissue to receive the implants. Each animal received two implants, one in each tibial metaphysis, one of each of the surfaces being evaluated. 

A total of 360 implants were placed, 180 with machined surfaces (Conexão, Arujá, Brazil) and 180 with fluoride-modified surfaces (Conexão, Arujá, Brazil). The implants presented 4 mm long and 2.2 mm in diameter. The preparation of the F surface was carried out by acid etching (H2SO4 + HCl + HNO3) followed by immersion of the implant in a solution containing Na^+^ and F^-^ ions (20).

An electric motor with a peristaltic pump (Osseocare®, Nobel Biocare®, Kloten, Switzerland) with a low-speed driller with 16: 1 reduction (Kavo®, Joinville, Brazil) was used for implant placement, following the operative sequence where it was used a progressive sequence of milling cutters (milling cutter, 2.0 mm spiral cutter - Connection®, Arujá, Brazil). The tissues were sutured using silk thread (Ethicon 4.0, Ethicon, Johnson Prod., São José dos Campos, Brazil) with interrupted stitches. The animals were medicated in the postoperative period with an association of penicillin and streptomycin (0.1ml/kg - Multibiótico®, Indústria Farmacêutica Vitalfarma Ltda, São Sebastião do Paraíso, Brazil) and analgesics (0.1ml/kg- Dipirona Ibasa 50%, Laboratório Ibasa LTDA, Porto Alegre, Brasil) with a single intramuscular injection. 

The animals were submitted to euthanasia with the application of anesthetic overdose at the periods of 2, 4, and 6 weeks after surgical procedures. It was placed 180 implants of each type at the total, of then, 90 implants were used for biomechanical analysis and the other 90 implants were used in the histometric evaluation.

### Biomechanical analysis

The biomechanical test was performed through the removal torque analysis. After the euthanasia of the animals, the implant platform was exposed and adapted to a prosthetic connector which enables the coupled of the digital torque wrench (TQ-680, Instrutherm, São Paulo, Brazil). With the aid of this torque wrench, an anti-clockwise movement was performed until the maximum torque peak force required for the bone/implant interface rupture was obtained and recorded for each implant that was removed.

### Histometry

For this analysis, the tibiae were removed and the implants and surrounding hard tissues were sectioned into blocks and placed in 4% buffered neutral formalin. After the fixation process (48 hours) and subsequent washing in running water, the specimens were dehydrated in an ethyl alcohol solution. The resin infiltration was carried out with mixtures of glycolmethacrylate (Technovit 7200 VLC, Kultzer Heraus GmbH & CO, Wehrheim, Germany) and ethyl alcohol, in increasing concentrations of resin, being in the end included in resin and polymerized.

The blocks were mounted on an acrylic sheet with the aid of the photopolymerizable resin (Technovit 7200 VLC, Kultzer Heraus GmbH & CO, Wehrheim, Germany). Using a micro-etching system (Exact-Cutting, System, Apparatebau Gmbh, Hamburg, Germany), the slides were processed to have a section of approximately 50 to 70 μm in thickness. Subsequently, the sections were stained by Stevenel's blue staining associated with acid fuchsin.

**Figure 2:**
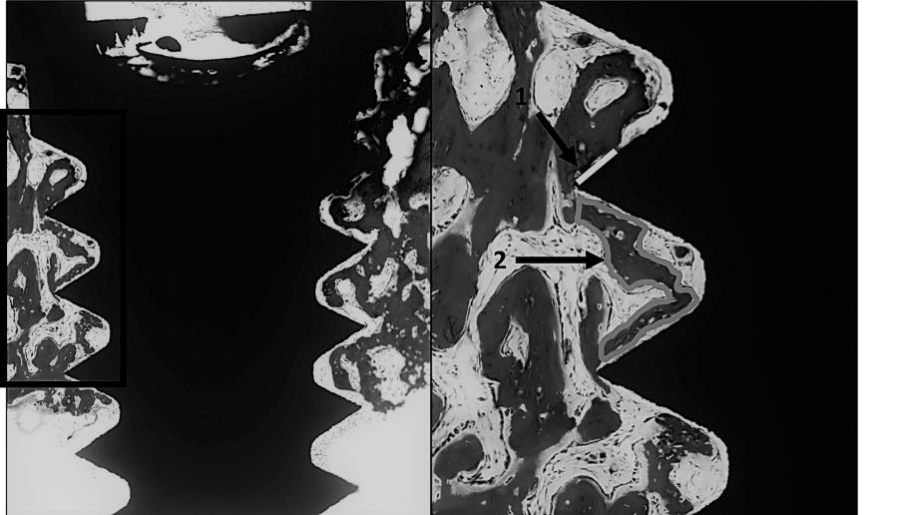
Description of the histomorphometric analysis. a) This analysis was performed at the first three threads inside the cortical bone; b) The analysis performed were: 1)The percentage of the linear direct contact of the bone and the implant surfaces (% BIC) and 2) The percentage of the fraction of the bone tissue area between the threads (% BBT)

To evaluate the osseointegration pattern, the following measurements were performed at the first 3 threads of the implants at the cortical bone area: 1) The percentage of the linear direct contact of the bone and the implant surfaces (% BIC) and 2) The percentage of the fraction of the ​​bone tissue area between the threads (% BBT) (Figure 2). The measurements were performed using a DM 2500 optical microscope (Leica Reichert & Jung products, Wetzlar, Germany), with a magnification of 100 X. Values were determined using an image analyzer software (Image J, San Rafael, CA, USA). These analyses were performed by a blind and trained examiner (LAF).

### Statistical analysis

The Jamovi software version 2.3.21 was used to perform the statistical analysis. The numerical data obtained of the body weight, glycemic level, biomechanical, and histometric analysis were submitted to the Shapiro-Wilk normality test to evaluate if the data were distributed according to the central distribution theorem. As the data were distributed according to the normality (p> 0.05), parametric tests were used for statistical analysis. The two-way Anova complemented by Tukey´s test was used to evaluate the data of the body weight and glycemic level associating the factors of glycemic condition and period of evaluation. The Three-way ANOVA complemented by Tukey´s test was used to evaluate the data of the biomechanical and histometric analysis associating the factors of glycemic condition, implant surfaces, and period of evaluation. All statistical tests were applied with a significance level of 5% (p <0.05). 

## Results

### Systemic data

**Figure 3:**
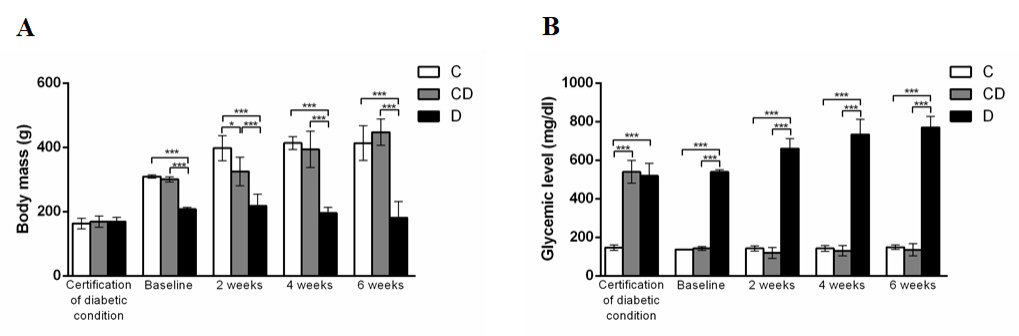
Representative graphics showing the mean and standard deviation of the a) body mass and b) glycemic level among the groups. *p<0.05; ***p<0.001 - Statistical differences between the groups - One-way ANOVA complemented by Tukey´s test.

All the animals survived after the surgical procedure and remained clinically stable during the whole experimental period. The animals of the D group presented the darkest and the least brightness hair and the cleaning of the boxes was performed more frequently due to the rapid accumulation of waste. On the day that the diabetic condition was certificated, animals from all groups presented statistically similar body weight. The glycemic condition and the experimental period influence the body weight (p<0.001). At the baseline and after 2, 4, and 6 weeks, rats from the D group showed statistically lower (p<0.0001) body weight when compared to C and CD rats. The body weight increased in the C and CD groups in longer experimental periods. (Figure 3). Regarding the glycemic levels, and proving the correct induction of the diabetic condition, on the day that the diabetic condition was certificated animals from the D and CD group showed statistically higher values when compared to C-group animals (p<0.0001). At baseline and also considering the 2-, 4- and 6-week periods of evaluation, the glycemic levels for the D group were always significantly higher (p<0.0001) than C and CD groups, which were always similar, proving the efficiency of the insulin therapy for CD group. The intra-group evaluation showed that, in the C group the glycemic levels remained constant during all experiments. In the CD group, the glycemic peak was achieved with the induction of the diabetic condition and was significantly reduced after the initiation of the insulin treatment. (Figure 3).

### Biomechanical analysis

Biomechanical data are shown in Table 1. The biomechanical data was statistically influenced by the experimental period, glycemic condition, and implant surfaces (p<0.001). The F surfaces improved the removal torque despite the systemic condition at 4- and 6-week periods of evaluation (p<0.001). The CD and C groups presented higher removal torque than the D group only associated with the F surfaces. These differences occurred during the 4 weeks (C vs. D) and the 6 weeks (C and CD group vs. D group) (p<0.05). The removal torque also increased in longer experimental periods associated with F surfaces except on D animals (p<0.05). 

**Table 1:** Biomechanical analysis data, expressed in means and standard deviations, according to group (C - control, CD - controlled diabetic, D - diabetic), period of evaluation, and implant surface (M - machined, F - fluoride treated).

Period	2 weeks		4 weeks		6 weeks	
Groups/Surfaces	M	F	M	F*	M	F*
C	1.53±0.73	3.71±0.55^b^	2.86±0.71	7.74±2.06^†*a^	3.79±0.78	11.78±1.87^†*a^
CD	1.46±0.56	3.80±1.6^b^	1.73±0.68	4.71±1.29*^b^	2.87±0.94	9.64±2.69^†*a^
D	1.06±0.33	2.49±0.81^b^	1.62±0.51	3.71±0.77^*b^	2.26±0.46	5.24±1.06^*a^

*Different from the M surface, in all groups (p<0.001); ^†^Different from the D group, in the same period of evaluation and considering the same surface (p<0.05); Different letters represent different levels of removal torque forces related with distinct experimental periods (p<0.05) - Three-way Anova complemented by Tukey test.

### %BIC and %BBT

**Figure 4:**
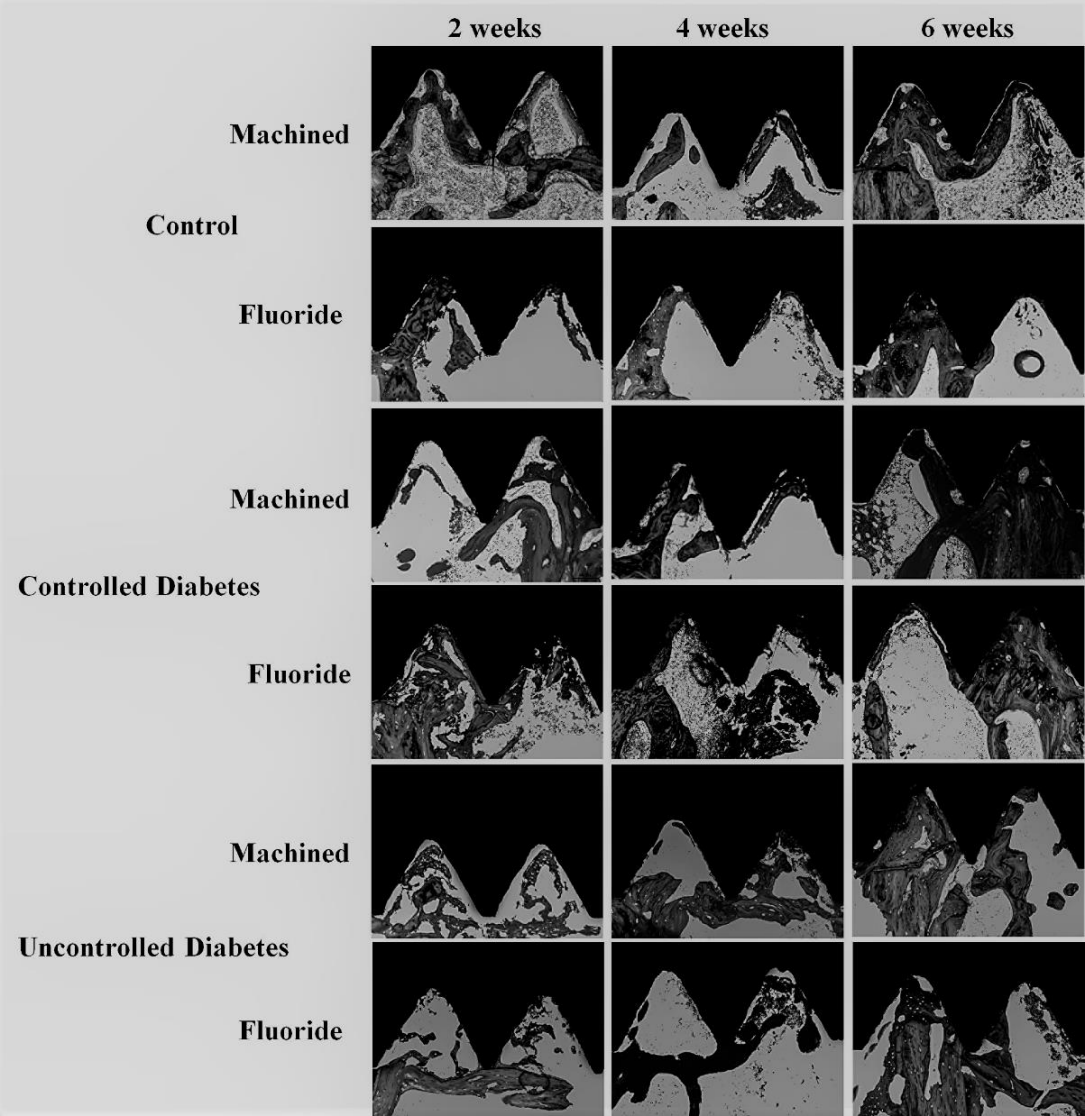
Representative histological images obtained by non-decalcified sections. It was observed a trend in the increasing of the osseointegration in longer evaluation periods. Despite the fluoride-modified implant surface improved the secondary stability in decompensated-induced diabetes, the histological aspect of the bone formation was quite similar between the implant surfaces.

The %BIC data was not influenced by the factors of implant surfaces and glycemic condition. There was an increase in %BIC at the 6-week periods compared with 2- and 4-week periods in all the groups except in F surfaces in C animals (p<0.05) (Table 2). The %BBT was not influenced by the experimental period and implant surfaces. The C and CD presented a higher %BBT than the D group at the 4 weeks (p<0.05). (Table 3). The representative images of the histometric analysis are shown in Figure 4.

**Table 2:** %BIC data, expressed in means and standard deviations, according to group (C - control, CD - controlled diabetic, D - diabetic), period of evaluation and implant surface (M - machined, F - fluoride treated)

Period	2 weeks		4 weeks		6 weeks	
Groups/Surfaces	M	F	M	F*	M	F*
C	27.40±16.65^b^	34.79±26.78	42.96±13.27^b^	44.04±12.75	55.68±22.27^a^	44.68±12.77
CD	35.73±15.49^b^	41.54±24.06^b^	33.46±13.06^b^	45.71±23.36^b^	50.71±22.60^a^	60.61±14.74^a^
D	32.89±17.86^b^	33.04±9.06^b^	36.11±17.82^b^	34.48±10.37^b^	49.55±14.80^a^	48.66±20.04^a^

**Table 3:** %BBT data, expressed in means and standard deviations, according to group (C - control, CD - controlled diabetic, D - diabetic), period of evaluation and implant surface (M - machined, F - fluoride treated)

Period	2 weeks		4 weeks		6 weeks	
Groups/Surfaces	M	F	M	F*	M	F*
C	36.45±15.16	25.98±9.65	41.22±22.84^†^	44.12±17.48	52.92±16.55	48.06±20.80
CD	38.05±24.58	22.49±11.84	55.04±12.26^†^	31.59±11.88	56.23±21.30	47.45±14.61
D	22.56±21.24	25.45±20.20	23.62±9.76	25.45±20.20	23.60±9.80	41.08±24.16

Different letters represent different levels of %BIC related with distinct experimental periods (p<0.05) - Three-way ANOVA complemented by Tukey test.

## Discussion

Fluoride use either in the form of subtraction modifications ^12^, ^20^, as used in this study, or by addition ^16^ has been considered one of the promising implant modifications. Regardless of the type of implant surface treatment with fluoride, these surfaces have been shown to potentiate the osseointegration process in preclinical models ^15^,^21^ and have promoted good clinical outcomes expressing high survival and success rates ^17^,^18^. Indeed, these previous findings were confirmed by this study since it was found that fluoride-modified surface implants had higher removal counter-torque results than turned implants in all groups of this study at 4 and 6-week periods.

Another finding of this study is that the implants placed in animals of the C and CD groups had higher values ​​of secondary stability than the implants placed in the animals of the D group associated with the F surfaces. These findings confirm the deleterious effects of hyperglycemia, which is characteristic of Diabetes mellitus, on the process of bone tissue metabolism ^22^,^23^, since this process is related to disturbances in the healing of connective tissues due to vascular changes, impaired protein maturation, and enhance the formation of AGEs that may culminate in the deficiency of bone tissue formation ^5^,^22^. Although the histometric analysis of %BIC did not show differences between the C, CD, and D, it is possible that the difference that occurred in the biomechanical analysis occurred due to the degree of bone maturation around the implants which directly influenced the establishment of secondary stability of the turned implants earlier in groups C and CD.

The use of fluoride-modified surface implants has promoted greater secondary stability of the implants installed in animals of the D group than that observed in machined implants placed in the animals of the CD groups. This fact is in agreement with the results of % BBT which demonstrated that fluoride-modified surface implants promoted an equality of these parameters between C, CD, and D groups, an event that was not observed when the implants installed had a machined surface. Indeed, in vitro studies have shown that this effect of fluoride-modified implant surfaces may be related to an increase in osteoblast proliferation ^12^, the increased expression of osteoblast differentiation markers such as Runx2 ^14^, the increased expression of markers of bone matrix synthesis such as type I collagen ^14^,^15^, and the higher expression of bone mineralization markers such as the osteocalcin ^14^,^15^. These stimuli were able to equalize the bone formation around the implants in the animals of the D group in comparison to the animals of the CD and C groups.

Some factors are important to be discussed regarding the implant surface tested and the studied population assessed. The implant surfaces tested in this study were morphologically modified by a subtractive treatment with fluoride, and then this ion was not incorporated into the implant surface as a coating ^20^. The F surface presented an alteration at the nanoscale level that has been indicated as the reason for its potential beneficial effect on the osseointegration process. However, improvements in this surface have been proposed since the cell viability was improved with the addition of bioactive molecules such as fibronectin ^20^. The effect of these molecules on fluoride-modified surfaces on osseointegration needs to be further investigated. Another important factor is the reason for investigating osseointegration in conditions of uncontrolled diabetes. The implant placement is indicated in controlled diabetic patients ^3^,^4^. However, it is important to admit that some patients who are controlled before the surgery can be uncontrolled during the osseointegration period since surgeries are one factor that can reduce the efficacy of the patients to control their glycaemic status ^8^. Another point to be considered is whether an implant surface can induce osseointegration in this challenging condition, maybe the contraindication for implant placement in the uncontrolled diabetes population can be reviewed. It is important to state that edentulism and the reduction of the occlusion efficacy may be considered as a risk factor for uncontrolled diabetes ^5^,^7^, and implant therapy can help these patients achieve appropriate glycaemic control.

Some concerns must be taken into consideration when evaluating the results of this study. One of these concerns is about the experimental model used for diabetes induction. The application of streptozotocin causes degradation of β-pancreatic cells which cause a reduction in insulin production and thereby mimics type 1 diabetes ^24^. Type I diabetes occurs less commonly than type 2 diabetes ^5^, and the effects of these different types of diabetes on bone tissue are different because the absence of insulin can also add deleterious effects to tissue ^5^ since this hormone is important in the metabolism of bone tissue ^25^. In fact, in this model, a reduction in the weight gain of the animals was observed, which was markedly lower than the animals of the C and CD groups and this fact may have influenced the process of bone remodeling in a more shocking way than the process of hyperglycemia, which can mean that the insulin absence is more important for bone healing and development than the deleterious effect of the hyperglycemia ^5^, ^24^, ^25^. Thus, the findings of this study may partially predict what may occur in patients with type 2 diabetes. Another limitation of this study is that implants that were compared to fluoride-modified surfaces had turned surfaces and it was not possible to identify if the improvements in the osseointegration induced by the fluoride-modified surface were also due to the surface roughness or mainly due to the different chemical composition of implants surfaces. However, this study provides important information that the fluoride-modified improves the pattern of osseointegration in a diabetic state, and to the best of our knowledge, this is the first study that evaluated the effect of fluoride-modified implant surfaces on the osseointegration in rats with controlled and uncontrolled diabetes. Finally, the analysis performed in this study was not able to evaluate the inflammatory pattern associated with osseointegration. This information is important to assess the inflammatory differences associated with osseointegration in hyperglycemic animals and if the fluoride-modified implant surface can modulate the inflammation during the osseointegration process. This effect may be evaluated in future studies. 

## Conclusion

The fluoride-modified implant surface has no impact on the %BIC and %BBT. However, the fluoride-modified implant surface increases the locking of the implants with the bone. The hyperglycemia was associated with lower removal torque values despite the surfaces of the implant used.
